# Incidence and clinical implications of ventricular tachycardia: a population-based approach

**DOI:** 10.1093/europace/euag234

**Published:** 2026-09-03

**Authors:** Peter Vibe Rasmussen, Anders Holt, Jacob Tønnesen, Charlotte Middelfart, Morten Lamberts, Jacob Tfelt-Hansen, Martin H Ruwald, Peter Karl Jacobsen, Morten Lock Hansen

**Affiliations:** Department of Cardiology, Rigshospitalet, Copenhagen, Denmark; Department of Cardiology, Herlev-Gentofte University Hospital, Copenhagen, Denmark; Department of Cardiology, Rigshospitalet, Copenhagen, Denmark; Department of Cardiology, Rigshospitalet, Copenhagen, Denmark; Department of Cardiology, Herlev-Gentofte University Hospital, Copenhagen, Denmark; Department of Cardiology, Rigshospitalet, Copenhagen, Denmark; Department of Cardiology, Rigshospitalet, Copenhagen, Denmark; Department of Cardiology, Rigshospitalet, Copenhagen, Denmark; Department of Cardiology, Rigshospitalet, Copenhagen, Denmark

**Keywords:** Ventricular tachycardia, Ablation, Anti-arrhythmic drugs

## Introduction

Ventricular tachycardia (VT) comprises arrhythmias originating from the ventricles, ranging from relatively benign conditions such as idiopathic non-sustained VT to sustained VT, which is a potentially life-threatening condition.^1^ Hence, VT is a major cause of cardiovascular morbidity and mortality in the general population and constitutes an important cause of sudden cardiac death.^2^

The pathophysiology of VT is multifactorial in nature and often categorized and treated according to the presence of underlying heart disease (e.g. ischaemic or structural heart disease).^3,4^ Albeit VT is considered important in terms of morbidity, mortality, and societal expenses, reliable population-based data regarding contemporary incidence and treatment are sparse outside the limitations of selected cohorts.^5^ Hence, using nationwide Danish data, we sought to investigate temporal trends in incidence, treatment patterns, as well as prognosis in patients with VT.

## Methods

In Denmark, it is possible to link multiple clinical registries enabling follow-up on an individual level (e.g. hospital diagnoses, surgical and interventional procedures, prescriptions dispensed at pharmacies). The registries have been described in detail elsewhere.^6–8^

First, an analysis was designed to describe temporal patterns in the incidence of new-onset VT using the whole adult Danish population as reference. Second, we constructed a cohort of adult patients (≥ 18 years and ≤100 years of age) defined by a first-time diagnosis of VT. Ventricular tachycardia was defined by first-time diagnoses comprising all types of monomorphic VT (including re-entry VT), idiopathic VT, as well as VT without further specification between 2011 and 2023.

The primary factors of interest were early catheter ablation, implantable cardioverter defibrillator treatment (ICD implantation), as well as pharmacological treatment with anti-arrhythmic drugs (AADs) and beta-blockers within 180 days (time of follow-up initiation). Anti-arrhythmic drugs in question were amiodarone, class 1c AADs, sotalol, and dronedarone. The clinical outcome of interest was all-cause mortality.

The annual incidence of VT [incidence rates (IR)] was calculated as the counts of incident diagnoses per 100 000 individuals per year during the study period (2011–23) and depicted graphically with 95% confidence intervals (95% CI). Temporal trends in the proportion of patients with therapeutic interventions (catheter ablation, ICD implantation, AADs) were depicted graphically. The absolute risk of all-cause mortality was calculated using the Aalen–Johansen estimator.

## Results

**Figure 1:**
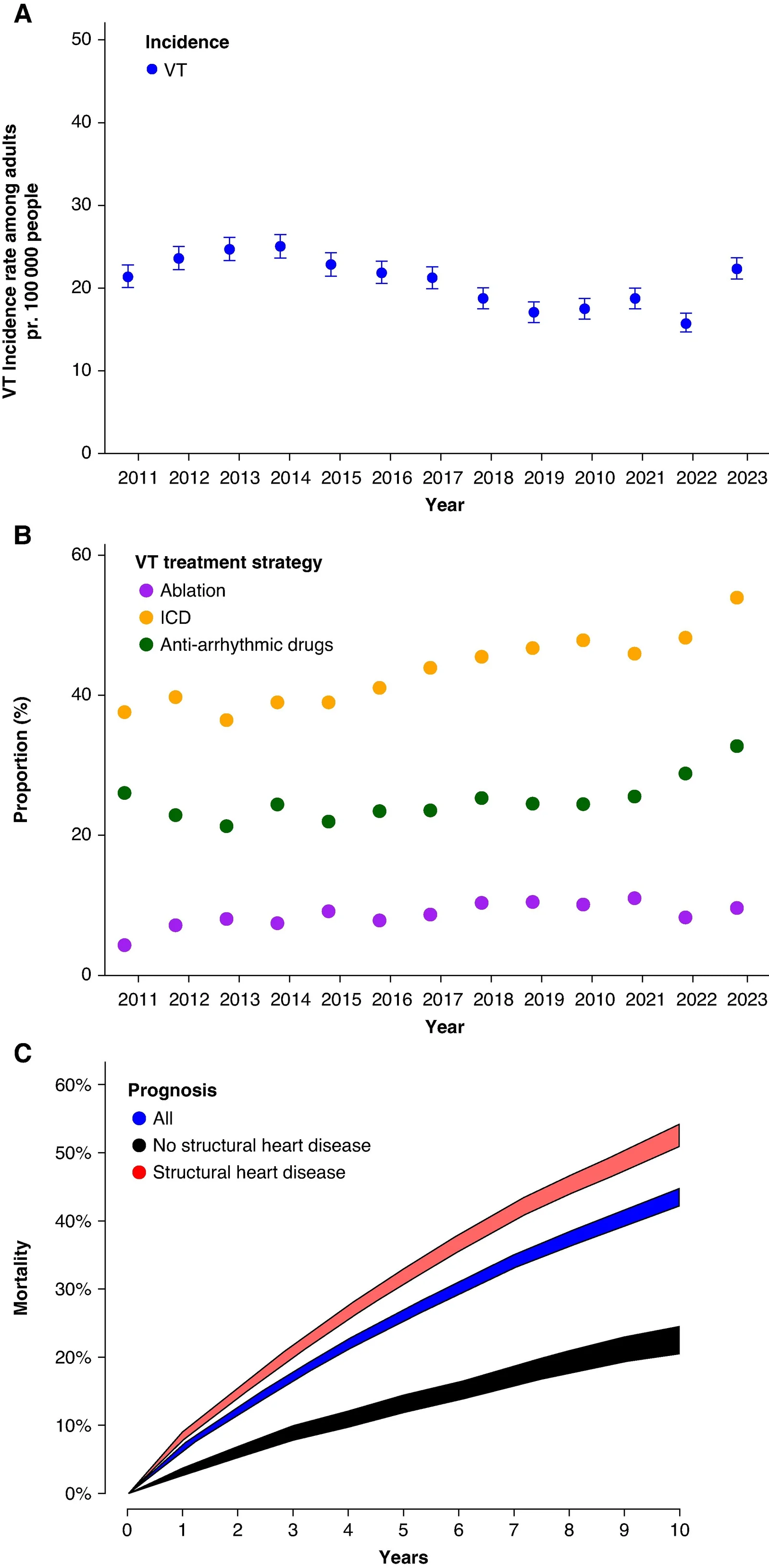
(*A*) Incidence rates of VT. The *x*-axis depicts years during the study period and the *y*-axis incidence per 100 000 individuals. (*B*) Figure depicting treatment strategies (ICD, ablation, and AADs). The *x*-axis depicts years during the study period and the *y*-axis the proportion in percentage (%). (*C*) The absolute risk of all-cause mortality in patients with VT. The blue colour depicts all VT patients, while the red and black colours depict VT patients with and without structural heart disease, respectively. The *x*-axis depicts time elapsed in years and the *y*-axis the risk of death in percentage (%).

The annual IR of VT remained relatively stable from 2011 until 2023 with a slight tendency towards decreased VT incidence over time (IR 21, 95% CI 20–23 per 100 000 individuals in 2011 to IR 17, 95% CI 16–18 per 100 000 individuals in 2023) (*Figure 1A*).

The prevalence of structural heart disease (including ischaemic heart disease) was substantial (69%), with significant ischaemic heart disease (56%), heart failure (44%), valvular heart disease (12%), as well as any diagnosis of a specific cardiomyopathy (17%).

The ICD implantation was common (43%) with an increase during the study period (38% in 2011 to 54% in 2023) (*Figure 1B*).

The use of AADs was common (25%), which remained relatively stable, albeit, with a small increase in the most contemporary years. The predominant AAD was amiodarone (24%). Beta-blocker treatment was very common (74%), which was unchanged during the study period.

Early catheter ablation increased during the study period (4% in 2011 to 10% in 2023) (*Figure 1B*).

Significant mortality was observed both in the short term (1-year mortality 6.8%, 95% CI 6.3–7.2), intermediate term (5-year mortality 26.1%, 95% CI 25.1–27.1), as well as long term (10-year mortality 43.3%, 95% CI 42.0–44.7) (*Figure 1C*). Moreover, the mortality associated with VT was highly dependent upon underlying structural heart disease.

During the study period (2011–23), we found no notable differences in the prognosis following a VT diagnosis as the mortality remained relatively stable (1-year mortality 6.6%, 95% CI 5.0–8.3 in 2012 compared with a 1-year mortality of 6.5%, 95% CI 4.6–8.5 in 2022).

## Discussion

Rhythm control interventions such as ICD implantation, catheter ablation, and AADs are all established interventions with proven clinical benefits and potential mortality reduction in patients with VT.^9,10^ Accordingly, these treatments were relatively common in our study with a trend towards increasing use in recent years. The frequent use of AADs, predominantly amiodarone (>30% in some years), could seem unexpected. Given the well-documented efficacy of catheter ablation in reducing VT burden, this could point to a gap between available evidence and contemporary clinical management strategies, as a proportion of patients could potentially benefit from early ablation rather than prolonged exposure to AADs with substantial side-effect profiles.^9^

The background for the modest adaptation of early ablation is unknown; however, patient selection, referral pathways, and accumulation of randomized evidence favouring early ablation published only in the later years of the study period could be hypothesized as important factors. Ventricular tachycardia was associated with significant mortality, especially in patients with structural or ischaemic heart disease (i.e. patients with more advanced myocardial substrate and more complex underlying disease), which were very prevalent in our cohort.

In this context, it is noteworthy that despite the broad clinical implementation of modern therapies, the overall prognosis in patients with VT was unchanged during our study period. However, this should not necessarily be interpreted as evidence that contemporary VT therapies are ineffective as ICDs primarily prevent arrhythmic death and catheter ablation reduces arrhythmia burden and ICD therapies. As such, the underlying myocardial disease and the risk of non-arrhythmic mortality remain unaffected. Moreover, changes over time in factors such as case mix and comorbidity burden could potentially have counterbalanced improvements in therapy.

Study limitations were generally inherent to the observational nature of the data with specific limitations such as no available electrocardiographic documentation to classify type of VT (e.g. VT exit site) or duration of VT episodes (sustained vs. non-sustained). Moreover, specific causes of death (i.e. arrhythmic vs. non-arrhythmic deaths) could not be ascertained from the available data.

Conclusively, we found an overall stable VT incidence over time with increasing adoption of ICD implantation and ablation; however, the frequency of early ablation remained modest. Mortality was significant and modified by the presence of structural heart disease, and importantly, the overall prognosis of VT in terms mortality did not change during the study period.

## Data Availability

Data for this study are derived from and accessed through Statistics Denmark and cannot be made publicly available by the authors as access is granted to research institutions on an individual basis by Statistics Denmark (https://www.dst.dk/da/TilSalg/Forskningsservice/Dataadgang).
